# Apps and Adolescents: A Systematic Review of Adolescents’ Use of Mobile Phone and Tablet Apps That Support Personal Management of Their Chronic or Long-Term Physical Conditions

**DOI:** 10.2196/jmir.5043

**Published:** 2015-12-23

**Authors:** Rabiya Majeed-Ariss, Eileen Baildam, Malcolm Campbell, Alice Chieng, Debbie Fallon, Andrew Hall, Janet E McDonagh, Simon R Stones, Wendy Thomson, Veronica Swallow

**Affiliations:** ^1^ University of Manchester School of Psychological Sciences Manchester United Kingdom; ^2^ Alder Hey Children’s Foundation National Health Service (NHS) Foundation Trust Department of Rheumatology Liverpool United Kingdom; ^3^ University of Manchester School of Nursing, Midwifery, and Social Work Manchester United Kingdom; ^4^ Royal Manchester Children’s Hospital Department of Rheumatology Manchester United Kingdom; ^5^ Arthritis Research UK Centre for Epidemiology Centre for Musculoskeletal Research University of Manchester Manchester United Kingdom; ^6^ National Institute for Health Research (NIHR) Manchester Musculoskeletal Biomedical Research Unit Manchester Academic Health Science Centre University of Manchester Manchester United Kingdom; ^7^ The University of Manchester Faculty of Life Sciences Manchester United Kingdom; ^8^ University of Leeds Leeds United Kingdom

**Keywords:** adolescents, asthma, mobile or tablet apps, arthritis, cancer, chronic disease or condition, diabetes, long-term condition, personal or self-management, review, young people

## Abstract

**Background:**

The prevalence of physical chronic or long-term conditions in adolescents aged 10-24 years is rising. Mobile phone and tablet mobile technologies featuring software program apps are widely used by these adolescents and their healthy peers for social networking or gaming. Apps are also used in health care to support personal condition management and they have considerable potential in this context. There is a growing body of literature on app use in health contexts, thereby making a systematic review of their effectiveness very timely.

**Objective:**

To systematically review the literature on the effectiveness of mobile apps designed to support adolescents’ management of their physical chronic or long-term conditions.

**Methods:**

We conducted a review of the English-language literature published since 2003 in five relevant bibliographical databases using key search terms. Two independent reviewers screened titles and abstracts using data extraction and quality assessment tools.

**Results:**

The search returned 1120 hits. Of the 19 eligible full-text papers, four met our review criteria, reporting one pilot randomized controlled trial and three pretest/post-test studies. Samples ranged from 4 to 18 participants, with a combined sample of 46 participants. The apps reported were targeted at type 1 diabetes, asthma, and cancer. Two papers provided data for calculating effect size. Heterogeneity in terms of study design, reported outcomes, follow-up times, participants’ ages, and health conditions prevented meta-analyses. There was variation in whether adolescents received guidance in using the app or were solely responsible for navigating the app. Three studies reported some level of patient involvement in app design, development, and/or evaluation. Health professional involvement in the modelling stages of apps was reported in all studies, although it was not always clear whether specific clinical (as opposed to academic) expertise in working with adolescents was represented. The dearth of studies and the small overall sample size emphasizes the need for future studies of the development, evaluation, use, and effectiveness of mobile apps to support adolescents’ personal management of their conditions.

**Conclusions:**

A key finding of the review is the paucity of evidence-based apps that exist, in contrast to the thousands of apps available on the app market that are not evidence-based or user or professional informed. Although we aimed to assess the effectiveness of apps, the dearth of studies meeting our criteria meant that we were unable to be conclusive in this regard. Based on the available evidence, apps may be considered feasible health interventions, but more studies involving larger sample sizes, and with patient and health professional input at all stages, are needed to determine apps’ acceptability and effectiveness. This review provides valuable findings and paves the way for future rigorous development and evaluation of health apps for adolescents with chronic or long-term conditions.

## Introduction

### Adolescents With Physical Long-Term or Chronic Conditions

Globally, the pattern of illness in young people or adolescents aged 10-24 years (hereafter referred to as adolescents) has shifted from acute to long-term or chronic conditions (hereafter referred to as chronic). A chronic condition in this age group is a health condition that at the time of diagnosis is predicted to last longer than 3 months [1]. At least 15% of adolescents aged 11-15 years report having been diagnosed with a chronic medical illness or disability [2]. Survival rates for this group have improved due to better screening, earlier detection, and improvements in the delivery of specialized care [1,3,4]. However, there is growing evidence to suggest that young people with chronic conditions have distinct health needs when compared to other groups [4,5].

Effective support from the health sector is therefore paramount, especially during the transition from pediatric to adult health services, and particularly if adult services are not young-person friendly [6]. This process of health transition as young people grow up requires them to develop clinical skills and knowledge in order to ultimately take responsibility for, and competently manage, their personal health care where appropriate [4,7-9]. Delivering safe and timely health care that is accessible and tailored to individuals’ needs and preferences is a central feature of international health care strategies [4]. Additionally, government policies highlight the need for services to support self-care; for example, the UK Department of Health and Department for Education are working to support young people with complex health needs in making the transition to adulthood [10].

Contemporaneous reports indicate that utilizing modern mobile electronic technologies in health interventions for young people [11-13] may be a suitable way to address self, shared, or joint care in a manner that is resource efficient.

Significant declines in treatment adherence have been observed during adolescence and the transition from pediatric to adult-centered health services [14]. Education interventions alone are insufficient to promote adherence, but outcomes could be enhanced by adding the following behavioral interventions: monitoring and goal setting, reinforcing medication taking with rewards, contingency contracting, problem solving, and linking medication taking with established routines [15,16]. However, the reported treatment effects are small and reflect the methodological limitations of the included studies and the need to re-examine the delivery and mechanisms of adherence-promoting interventions.

In a recent commentary, Wu and Hommel [17] describe current and potential technologies, such as short message service (SMS) text messaging, mobile phone apps, electronic monitors of adherence, and illness-specific medical devices, to promote pediatric adherence to prescribed medical regimens. The uses reported include the following: delivering and collecting information, facilitating communication between patients and professionals, social networking, capturing real-time data, monitoring bodily functions, automated feedback, guidance and clinical alerts, and smart decision-making tools. However, despite the significant potential and increased use of mobile technologies, to our knowledge there has not been a synthesis of studies reporting on their effectiveness in the management of physical chronic health conditions in adolescents.

### Mobile Phone and Tablet Apps to Support Chronic Condition Management

Personal management of chronic physical conditions involves five core skills: problem solving, decision making, resource utilization, forming patient-health professional relationships, and taking action [18]. Apps can support these skills through knowledge development and by providing and collecting information in an accessible, convenient, and interactive way. Mobile phones and tablets form the new generation of mobile electronic devices, different to previous generations in that they are a consumer product as opposed to primarily a business product [19]. Mobile phones and tablets can function with custom software programs called apps, which technologically allow the development of condition-specific and patient-tailored software. These are personal devices, adapted by the user to reflect their specific needs, thus allowing for adaptive, responsive, confidential, and targeted channels of communication and alerts.

A review of the effectiveness of mobile health technology-based health behavior change or disease management interventions for adults found that only six of the 49 interventions used apps and none of these involved adolescents [12]. Another review of mobile phone interventions for management of chronic disease in 18-73-year-olds [20] found few mobile apps and recommended that more be developed. In the Italian health care Android market the majority of apps were designed for health care professionals [21]. Since the potential of mobile technologies in personal health care is significant, a growing body of literature on the use of apps to support patients’ management of chronic conditions is emerging.

Mobile apps are widely accepted by adolescents living in today’s technology-rich environment. In the United Kingdom for instance, children and adolescents aged 5-15 years are frequent users of mobile technologies. Indeed, 62% of 12-15-year-olds own a mobile phone, and the use of tablet computers by 5-15-year-olds tripled between 2012 and 2013 with 42% using tablets in 2013 [19]; these upward trends are expected to continue. Mobile technologies offer new opportunities to engage adolescents in personal health care [4] but are not without their challenges.

In 2013, the UK National Health Service (NHS) Commissioning Board unveiled a library of NHS-reviewed health apps [22]. Although the review focused on clinical safety rather than clinical effectiveness, it acknowledged that the computing capability contained within mobile technologies offers a legitimate platform for medical and public health practice. However, the Institute of Medical Science (IMS) Institute of Healthcare Informatics (IMS Health) [23] reported that the lack of evidence regarding the effectiveness of mobile apps acts as a barrier to physicians prescribing them. The IMS identified a pressing need for credible evidence of the value of health apps, which in many cases are being used without a thorough understanding of their associated risks and benefits, or a rigorous, evidence-based approach to their development and evaluation [24].

Yet despite increased use and the significant potential of these technologies for adolescents with chronic conditions, to our knowledge a synthesis of studies of their effectiveness in this population has not been undertaken. This systematic review of the evidence is, therefore, timely as it aimed to assess the effectiveness of mobile phone and tablet apps for adolescents’ personal management of chronic conditions. In this review, young people are defined as those aged 10-24 years (as defined by the World Health Organization [WHO] [25]) who are undergoing key elements of development, particularly brain development, which continues until the early 20s [5,25,26]. This is arguably a crucial time for the introduction of interventions that promote shared and self-management skills and knowledge, and for the development of both health-promoting as well as health-risk behaviors. The review protocol was published previously in JMIR Research Protocols [27], but key details are reiterated here for new readers.

## Methods

### The Systematic Review

This systematic review aimed to synthesize the evidence on mobile phone and tablet apps. The methodology adhered to that described in the Cochrane Handbook for Systematic Reviews of Intervention [28] and complies with the Preferred Reporting Items for Systematic Reviews and Meta-Analyses (PRISMA) guidelines [29]. This review was registered with the international prospective register of systematic reviews (PROSPERO) (CRD42014015418) [30].

### Search Strategy

Eligible studies were identified through a comprehensive literature search of the following five bibliographical databases: MEDLINE, the Cumulative Index to Nursing and Allied Health Literature (CINAHL), Embase, PsycINFO, and the Web of Science. The search strategy, which was developed in consultation with an information scientist, used standardized indexed search terms and free-text terms that relate to the following three key concepts: (1) adolescents, (2) physical chronic conditions, and (3) mobile technology. The search included British and North American terms and spellings. The search strategy was initially devised in MEDLINE and then adapted to the other databases. The Web of Science did not employ any indexed search terms and the other databases did not employ them in a standardized fashion. Free-text terms were used consistently throughout. In addition to testing search sensitivity, journals associated with the most retrieved citations were hand searched from 2009 to 2014, thus decreasing the likelihood of missing relevant studies. The identification of any studies additional to those we had identified from hand searching allowed us to comment on the rigor of the search strategy and the quality of indexing in the bibliographic databases mentioned above. This is a particularly useful strategy in this relatively new domain of mobile technology. Also, due to the emerging nature of mobile technology, the search included conference abstracts published in peer-reviewed journals, and authors were contacted requesting additional related published or unpublished work.

### Screening and Selection Criteria

#### Overview

Initially, all papers were independently scrutinized by two reviewers (MR, AH) using a screening tool that outlined the study inclusion criteria (see Textbox 1). The 782 articles that met this criteria were then divided between two teams of two reviewers—Team 1: MR, VS and Team 2: AH, DF—who further scrutinized the abstracts using the same screening tool. Whenever disagreement in interpretation arose within one team, the other team was asked to consult the relevant materials to enable a discussion until a consensus between both teams was reached, thereby minimizing bias in the interpretation of findings. Team meetings were held regularly to discuss any complications or challenges.

#### Inclusion Criteria

Criteria for included studies are shown in Textbox 1.

**Summary of inclusion criteria.**

Inclusion criteria

- Population: Adolescents aged 10-24 years (WHO definition from 2001 [25]) diagnosed with chronic physical conditions in any setting.
- Intervention: Any app for a mobile phone or tablet that could be considered a management intervention (or a component of an intervention) in terms of content and/or delivery. This judgment was based on the five core management skills for chronic physical health conditions, as outlined by Lorig [18].
- Comparisons: Intervention versus usual care *or* intervention variant versus intervention variant *or* pre and post.
- Outcomes: Any physiological, attitudinal, behavioral, or knowledge outcomes.
- Study design: Randomized controlled trial (RCT) *or* controlled clinical trial *or* cohort analytic study *or* case-control study *or* cohort study *or* interrupted time series.

The Cochrane Collaboration excludes nonrandomized controlled trials due to their greater bias, but because this is a relatively new area, we included studies of various designs to systematically collect an overview of the current evidence.

#### Exclusion Criteria

While international literature was included, non-English-language publications and studies that focused on adolescents with mental health problems, learning disabilities, and/or cognitive impairment were excluded due to resource limitations. Interventions using mobile phone technology only in the context of delivering/receiving text messages or phone calls were also excluded. Given the review focus, the technology context was considered key so we applied a publication start date of 2003. This is the year when 3G networks, which provided the bandwidth required to support advanced mobile Internet apps, were launched in the United Kingdom [31]. By January 2007, 147 wideband code-division multiple access (WCDMA)—the standard found in 3G mobile telecommunications—network operators were delivering commercial services to over 100 million subscribers in 67 countries on all continents [32].

### Data Extraction

For every included study, two reviewers extracted relevant data independently. A tool based on the data extraction template for Cochrane reviews [33] was developed to facilitate consistent data extraction and prevent important information from being overlooked. Any discrepancies between reviewers were resolved by discussion with the wider research team. Where required, authors were contacted for clarification or additional information. Completed electronic extraction sheets were kept as part of the audit trail, should they be required at a later stage to enable data checking.

### Quality Assessment

The evidence and quality of the papers included in the systematic review were assessed using the Effective Public Health Practice Project (EPHPP) quality assessment tool [34,35]. This method requires a review team with at least one member having methodological expertise, and two members with subject expertise; the team for this review met these criteria. The tool involves six component rating domains: selection bias, study design, confounders, blinding, data collection methods, and withdrawals and dropouts. As with the data extraction stage, each study was scored independently by two reviewers, and any disagreements were resolved through discussion with the wider team.

### Data Synthesis and Inter-rater Reliability

Except where indicated, extracted data from the papers were analyzed using IBM SPSS Statistics version 22.0 (IBM Corp). For each paper, inter-rater reliability was estimated for each of the domains, the total score, and the final grade of the EPHPP. Following Armijo-Olivo et al [35], agreement for each domain and the final grade before consensus was estimated using Cohen’s unweighted kappa statistic (κ) [36]. Values were interpreted using the criteria of Altman [36]: κ>.80 was interpreted as very good, .61-.80 good, .41-.60 moderate, .21-.40 fair, and ≤.20 poor agreement. Because the scores for each domain and the final grade were ordinal (1=strong, 2=moderate, 3=weak), Cohen’s weighted kappa was also estimated [36]. Unweighted and weighted kappas and their 95% confidence intervals were estimated using an online calculator on the VassarStats website [37]. Again following Armijo-Olivo et al [35], inter-rater agreement on the overall score across the domains was estimated using an intraclass correlation coefficient (ICC), using a two-way, mixed-model analysis of variance with assessor as the fixed factor and paper as the random factor. Values of the ICC were interpreted against the criteria recommended by Armijo-Olivo et al: ICC≥.75 was interpreted as excellent, .60-.74 good, .40-.59 fair-to-moderate, and ≤.40 poor agreement. Fleiss and Cohen [38] demonstrated the equivalence of weighted kappa and the ICC, so these criteria were also used to interpret weighted kappa.

## Results

### Study Selection

The combined electronic searches identified 1120 records. Of these, 338 records were removed after accounting for duplicates, leaving 782 records for further consideration. Out of the 782 titles and abstracts that were then screened to test eligibility using the PICOS screening tool (population or participant, intervention or indicator, comparator or control, outcome, and study design), 19 full-text papers were potentially eligible for inclusion. Many of the excluded papers reported observational, noncontrolled studies that did not focus on the population or intervention of interest.

**Figure 1:**
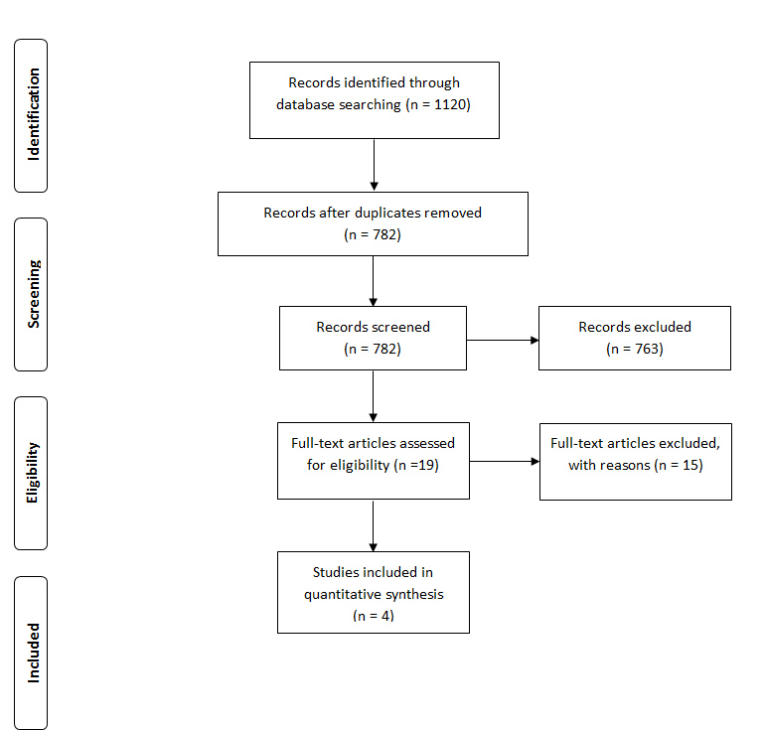
PRISMA flow diagram of the review.

Seven of the 19 studies included from the title and abstract screen were abstracts from conference presentations; a search was undertaken to find the full paper of each abstract, failing which the corresponding author was contacted. For two of the seven conference papers, we found subsequent publications [39,40]. We contacted the remaining five authors; three responded with information that meant we were able to exclude their work from the review, and two did not respond meaning their abstracts were also excluded as the full paper was unavailable for consideration in the review. Of the 19 full-text papers considered for eligibility, four papers were assessed as suitable for the full review. Multimedia Appendix 1 lists the respective reasons for excluding the remaining 15 papers [40-54]. Figure 1 illustrates the PRISMA flowchart representing the study selection process. Further hand searching of the Journal of Medical Internet Research from 2009 to 2014 did not identify any additional studies.

### Characteristics of Included Studies

#### Overview

The four included studies described four different apps addressing the needs of adolescents with different chronic conditions: type 1 diabetes [55,56], asthma [39], and cancer [57]. The commonality among all apps studied was that they aimed to support the adolescent in the medical management of their physical condition. Table 1 provides an overview of the four studies. Three of the studies were pretest/post-test designs with no control group [39,55,56] and one was a pilot RCT [57], which used a variety of measurements [58-60]. Two of the studies were conducted in North America [39,55] and two in Western Europe [56,57]. All of the studies recruited adolescents from secondary health care, and follow-up times ranged from 2 to 12 weeks. Based on the data provided, it is not possible to comment on mean age, gender, or ethnicity of the overall sample. The sample sizes ranged from 4 to 18 participants, with a combined total sample of 46 participants. Since the included papers were reporting on feasibility studies with small sample sizes, generalizability of the findings cannot be commented on.

Aldiss et al evaluated the Advanced Symptom Management System (ASyMS) which utilizes mobile phone technology to monitor chemotherapy-related symptoms and promote self-care [57]. This system was first developed with an adult cancer population but Aldiss et al are using a three-phased approach to adapt it for use by adolescents (or young people) with cancer (ASyMS-YG). Phase 1 had involved adolescents identifying the symptoms to be assessed; in Phase 2 adolescents tested the symptom report system, and adolescents’, parents’, and professionals’ perceptions of ASyMS-YG were ascertained [61,62]. The paper included in this review reports on Phase 3 in which the system was developed further in preparation for an RCT. Aldiss et al evaluated it with a group of adolescents with cancer, asking them to complete the ASyMS questionnaire once a day for 14 days during a cycle of chemotherapy.

**Table 1:** Characteristics of the included studies.

Study	Country	SS^a^, n	Measurements	ID^b^, weeks	F^c^, %	Age (years), mean (range)	Study design	Cond.^d^
Aldiss et al [57]	UK	4	Pediatric Quality of Life Inventory Cancer Module Teen Report Form [58] The Life Situation Scale for Adolescents (LSS-A) [59] The State-Trait Anxiety Inventory (STAI) [60] Perceptions of Technology Questionnaire	2	0	N/A^e^ (13-15)	Pilot RCT^f^	Cancer
Burbank et al [39]	USA	18	Self-efficacy questionnaire Asthma Control Test	8	N/A	13.5 (12-17)	Feasibility pretest/ post-test	Asthma
Cafazzo et al [55]	Canada	12	Self-care inventory Diabetes Family Responsibility Questionnaire Diabetes QOL^g^Instrument for Youth	12	67	14.9 (12-16)	Pretest/ post-test (mixed method)	Type 1 diabetes
Frøisland et al [56]	Norway	12	HbA1c^h^ Knowledge test score, before and after the intervention System Usability Scale after the intervention	12	54	16.2 (13-19)	Pretest/ post-test (mixed method)	Type 1 diabetes

^a^SS: sample size.

^b^ID: intervention duration.

^c^F: female.

^d^Cond.: condition.

^e^N/A: not applicable (information not reported or stated).

^f^RCT: randomized controlled trial.

^g^QOL: quality of life.

^h^HbA1c: hemoglobin A1c (glycated hemoglobin).

In light of the national asthma education program recommendation for a written asthma action plan (AAP) for all patients with asthma, and in recognition that few studies demonstrating acceptance of phone-based self-monitoring have taken place in rural and medically underserved US regions, Burbank et al tested a mobile AAP mobile phone app for adolescents with persistent asthma in Arkansas [39]. The app was designed to help self-monitoring by recording behaviors as well as prompting positive behaviors by providing immediate feedback on data entered.

Cafazzo et al [55] designed an mHealth intervention for the management of type 1 diabetes in adolescents that aimed to increase the frequency of daily blood glucose readings and to assist with self-care behaviors, establishing the use of technology to improve glycemic control among adolescents as a long-term objective [55]. Addressing the adolescent’s need for fast, discrete transfer of blood glucose data, this team developed the bluglu adapter to facilitate automated data transfers (via Bluetooth) from a glucometer to an iPhone or iPad touch device running the bant app; the app then analyzes the data to facilitate feedback to the adolescents in real time. Actions were rewarded with iTunes and apps, which introduced the notion of gamification to this intervention. During a 12-week evaluation, 20 diabetic adolescents aged 12-16 years were supplied with the bant app, glucometer, and bluglu. The outcome measure was the average daily frequency of blood glucose measurement during the pilot compared with the preceding 12 weeks. Finally, Frøisland et al [56] tested adolescents’ experiences with a diabetes diary known as Diamob, which recorded data before a mandatory consultation with a health professional to discuss the app and reflect on its recordings.

#### Effect Sizes

Where possible, Cohen’s *d* statistic [63] was calculated as an intention-to-treat effect size for outcome measures in each paper following Donker et al [64]. The one study that was a randomized controlled trial [57] did not report any quantitative results as only 3 of the 4 participants completed the trial. The only papers where estimation of effect sizes was possible were pretest/post-test designs, so Cohen’s *d* was calculated for each measure as mean post-test score minus mean pretest score divided by the pooled standard deviation [65]. While this approach does not take into account the repeated nature of the data, the alternative approach to divide the mean difference by the standard deviation of the difference score [65] requires statistical information that is not routinely published. The analysis in one paper [39] was nonparametric, but the authors did not explain why; descriptive statistics for outcomes were reported as medians and interquartile ranges, which were insufficient for reliable estimation of Cohen’s *d*. Cohen’s *d* was estimated for the remaining two papers using a Microsoft Excel spreadsheet from Missouri State University [66]. Cohen’s *d* was interpreted using Cohen's own criteria [63]: 0.80 was interpreted as large, 0.50 as medium, and 0.20 as small.

Cafazzo et al recruited adolescents aged 12-16 years who were diagnosed with type 1 diabetes for more than 1 year, were receiving care at one clinic for at least 6 months, had a hemoglobin A1c (HbA1c) level between 8 and 10%, and were able to communicate in English [55]. Frøisland et al recruited 13-19-year-olds who were diagnosed with type 1 diabetes for more than 1 year, were receiving care at one of two pediatric clinics, and had an HbA1c level of less than 10% [56]. Aldiss et al [57] and Burbank et al [39] did not provide information regarding participant selection and inclusion criteria other than their selection of adolescents with the named chronic condition.

### The Interventions

All four apps focused on management of a chronic condition. Cafazzo et al’s [55] and Frøisland et al’s [56] apps for type 1 diabetes management focused on increasing the number of blood glucose readings. Burbank et al developed an app outlining an AAP to improve asthma management [39]. These three apps can be seen as aids to prevent deterioration of the respective conditions, and in this sense are different from the app delivered by Aldiss et al, which was oriented toward recovery and improved chemotherapy experience [57]. As this difference in delivery time in terms of the different stages in the care pathway demonstrates, the apps described in the included papers address the needs of adolescents with various conditions. These conditions have similarities as well as notable differences. Moreover, there is heterogeneity in the content and delivery of these apps.

There was variation in whether apps were delivered as a stand-alone resource or whether they were used alongside other components of a medical intervention. Burbank et al [39] and Cafazzo et al [55] delivered the app as a stand-alone resource. In the case of the app developed by Aldiss et al, hospital-based nurses were alerted if there was cause for concern; adolescents could also make phone calls to the hospital if they wished [57]. The Frøisland et al study involved a consultation with a health professional midway through the intervention period to give the adolescent an opportunity to discuss and reflect on their use of the app [56].

There was also variation in whether adolescents received guidance in using the app or whether they were solely responsible for navigating the app. Aldiss et al [57] and Burbank et al [39] delivered their apps so that adolescents had sole responsibility in their navigation. Cafazzo et al [55] and Frøisland et al [56] spoke of the adolescents receiving initial training in using the app and the model of mobile phone that would be used in the intervention. The studies also differed in regard to reporting a primary outcome measure; Aldiss et al included six psychometric measures, although they did not identify a primary outcome measure [57]. Cafazzo et al described their primary outcome as an increased frequency of daily blood glucose readings [55]. Frøisland et al simply listed outcomes in the following order: HbA1c, system usability, and knowledge [56]. Burbank et al listed the following outcomes: usage and satisfaction rates, asthma control test, and asthma self-efficacy [39]. One app—developed by Cafazzo et al [55]—was underpinned by the concept of ecological momentary interventions [67], while the other apps were not theoretically driven.

### Quality Assessment

Table 2 reports the score on the six individual quality assessment items as scored by the EPHPP tool [34].

**Table 2:** Study quality assessment for each study.

Quality assessment items	Study and quality assessment score^a^			
	Aldiss [57]	Burbank [39]	Cafazzo [55]	Frøisland [56]
Selection bias	Weak	Weak	Weak	Weak
Study design	Moderate	Weak	Moderate	Weak
Confounders	Weak	Weak	Weak	Weak
Blinding	Moderate	Weak	Weak	Weak
Data collection method	Moderate	Strong	Strong	Weak
Withdrawals and dropouts	Moderate	Strong	Moderate	Strong

^a^Items were scored using the Effective Public Health Practice Project (EPHPP) quality assessment tool.

### Inter-rater Reliability

Across the four papers, there was moderate-to-good agreement between the raters on the domains of the EPHPP quality assessment tool, with agreement on 17 of the 24 domains (71% agreement; unweighted κ = .60, 95% CI .33-.87; weighted κ = .71, 95% CI .49-.93). There was excellent agreement on the total domain scores for the four papers (ICC = .83, 95% CI -.17 to .99). The wide confidence intervals reflected the small number of papers assessed.

### Effect Sizes

Only two papers provided data for calculating effect sizes and both featured mobile phone apps to help adolescents with type 1 diabetes. Both papers reported small sample sizes; Cafazzo et al [55] reported outcome data for 14-20 participants while Frøisland et al [56] reported data for 11-12 participants, and as these were feasibility studies, they would not have been powered to detect statistical significance. Cafazzo et al reported means and standard deviations before and after their intervention for a wide range of outcome measures [55], but did not report standard deviations for their primary outcome of frequency of blood glucose measurement that showed a significant improvement (*P*=.006), so it was not possible to estimate Cohen’s *d*. The change in HbA1c level was numerically small—from 8.8 to 9.2—and nonsignificant, but the effect size was good (*d*=-0.46, *P*=.11). They found no significant changes in dimensions of the Diabetes Self-Care Inventory and the effect sizes varied (adherence *d*=0.11, blood glucose regulation *d*=0, insulin and food regulation *d*=0.12, and emergency preparedness *d*=0). For the only dimension where there was a near significant but sizeable improvement (exercise *d*=0.56, *P*=.07), Cafazzo et al attributed the improvement to a seasonal change from winter to spring as their intervention did not target exercise [55]. While they found no significant improvements in dimensions of the Diabetes Family Responsibility Questionnaire, the effect sizes were medium to large (caregiver’s perspective *d*=0.69 and adolescent’s perspective *d*=0.72); however, the mean scores only changed by 0.1 point on a 3-point scale. They also found no significant changes in dimensions of the Diabetes Quality-of-Life Instrument for Youth, where the effect sizes were small or small to medium (impact of symptoms *d*=0.36, impact of treatment *d*=-0.07, impact of activities *d*=0.26, parent issues *d*=0.16, worries about diabetes *d*=0.30, and health perception *d*=0.15). Frøisland et al reported means and standard deviations before and after their intervention for HbA1c levels and a knowledge test score [56]. Neither outcome showed a significant difference nor an effect size better than small (HbA1c mean *before* 8.3 [SD 0.9], mean *after* 8.1 [SD 0.9], *d*=0.23, *P*=.38; knowledge test *d*=0.04, *P*=.82).

### Patient and Public Involvement in the Included Studies

Since mobile phones and tablets as the new generation of mobile electronic devices are a consumer product with custom software programs called apps, it is perhaps especially important to take into account adolescents’ attitudes toward using apps developed to help manage their chronic conditions. While we know mobile apps in general are widely accepted by adolescents living in today’s technology-rich environment, we were interested to know whether adolescents had been involved in the development of apps included in this review that were aimed at them and their peers. Three of the four studies we reviewed reported some level of patient involvement in the design, development, and/or evaluation of the app (see Table 3). These three papers reported using qualitative research methods for informing the development of mobile apps for adolescents with cancer [57] and adolescents with type 1 diabetes mellitus [55,56].

**Table 3:** Involvement of adolescents and their families at different stages of mobile app design, development, and evaluation.

Study	Involvement of adolescents and families at each stage		
	Design	Development	Evaluation
Aldiss et al [57]	Identified the symptoms to be assessed and addressed self-care advice	Tested the symptom report system	Reported perceptions of intervention and reviewed the self-care advice Procedures and technical systems were assessed
Burbank et al [39]	Not reported	Not reported	Not reported
Cafazzo et al [55]	Requirements were obtained through qualitative interviews and focus group sessions	Themes were derived from focus group sessions, which were incorporated into the prototype app	Not reported
Frøisland et al [56]	Adolescents suggested improvements for a future app	Not reported	Technical problems were reported, along with improvements of the existing app

Adolescents were important contributors in the development of ASyMS, an advanced symptom management system utilizing mobile phone technology for adolescents with cancer [57]. The authors reported that during Phase 1 development, adolescents’ contributions were essential in identifying which symptoms should be assessed via the ASyMS questionnaire. In Phase 2, adolescents tested the symptom report system. As the software progressed into Phase 3 development, adolescents aged 13-18 years who were receiving chemotherapy were involved in addressing and reviewing the self-care advice. Interestingly, these adolescents suggested improvements to the questionnaire by adding descriptive indicators to clarify specific aspects of the intervention; these had not been identified as areas for improvement by researchers.

Similarly, in Cafazzo et al’s pilot study [55], 6 adolescents and their parents informed the design and development of a mobile app to support adolescent self-management of type 1 diabetes. Focus groups with adolescents and their parents revealed specific requirements that were expressed as four themes: the need for fast, discrete transactions; the role of data collection rather than decision making; overcoming decision-making inertia; and ad hoc information sharing. These opinions were incorporated into the prototype version of the app for testing.

Frøisland et al [56] also involved adolescents in the redesign and evaluation of a mobile visual learning intervention for adolescents with type 1 diabetes, whereby they provided guidance for further development of the mobile app. In addition, adolescents also suggested improvements for the existing app, with requests for additional functionality. The authors reported their intention to implement the adolescents’ suggestions when designing the next version of the app.

### Clinician Expert Involvement in the Included Studies

Appropriate clinical expertise in the specific condition was evident in all four of the research teams, although it was not always clear whether specific clinical (as opposed to academic) expertise in working with adolescents was represented. Health professional involvement in the modelling stages was reported in three of the four studies [39,55,57]. The study by Aldiss et al was exemplary in view of the meaningful involvement of both adolescents and professionals in the study; in particular, adolescents with specific expertise were involved in the modelling stages of development [57]. Although Cafazzo et al conducted focus groups with health professionals in the modelling stages, no results were presented [55]. In the one study which reported theoretical underpinning [55], involvement of health professionals in translating this into the modelling stage was not reported. In the Frøisland et al paper, it is not clear whether the health professionals had any specific input into the app development [56]. However, the finding that the adolescents’ theoretical clinical knowledge was not altered after use of the app demonstrates that the intentions of the app use needs to be linked to a system of assessing its efficacy.

## Discussion

### Principal Findings

This review clearly demonstrates that despite the large number of health care apps in existence, the evidence base for their benefits for adolescents in personal management of their chronic physical health conditions is limited. The additional contextual challenge is that manufacturers are readily developing apps which are not based on empirical evidence [68]. Studies included in this review were all in the early proof-of-concept phase with few participants, meaning that assumptions about generalizability of the findings to the target population cannot be made. The findings reported are therefore preliminary and would need to be validated by larger-scale research. Comparisons between studies are also difficult as a result of the variability including the short and different follow-up times. While these studies alone do not provide high levels of evidence, they do provide valuable information that paves the way for other studies to inform future development and evaluation of complex app interventions [69].

### Adolescent-Specific Issues

As increasing numbers of adolescents with chronic conditions have transferred to adult-centered care, significant declines in treatment adherence have been observed during adolescence and the transition period [70]. Using educational interventions alone to enhance medication adherence is insufficient, but the addition of behavioral elements, such as monitoring and goal setting, rewards, contingency contracting, problem solving, and linking medication taking with established routines, may enhance outcomes [15,71]. That said, the small treatment effects of recent adherence-promoting interventions reflect the need to re-examine their delivery, and the mechanisms of emotional, social, and family processes [72]. Adolescence is arguably a crucial time for the rigorous development, evaluation, and implementation of interventions that promote shared and self-management skills and knowledge, and for the promotion of healthy behaviors [73]. While it is widely recognized that communication technologies are important drivers in adolescent health [4], there are barriers to the use of mobile technologies by adolescents. These include the disparity of access to mobile devices and the potential for habituation, suggesting that the use of information technology (IT) to address health issues may be limited or even harmful to adolescents [74,75].

### Participatory Design

Although these studies [39,55-57] support the view that engaging adolescents with chronic conditions has contributed to changes in mobile intervention design, the effects of involvement on accessibility and acceptability (ie, retention and use of mobile apps) was not examined. A consistent finding in this systematic review suggests that adolescents are engaged in helping to design mobile interventions; however, they may not have been actively involved as equal contributing partners in the entire research development and implementation process, as recommended by INVOLVE, the public involvement body funded by the UK National Institute for Health Research. INVOLVE suggests that involvement, engagement, and participation are often linked, and although they are distinct roles, they can indeed complement each other [76]. Examples of patient and public involvement in research include coapplicant responsibility on grants and research projects, involvement in identifying research priorities, membership of project steering groups, and undertaking interviews with research participants [77]. The meaningful involvement, engagement, and participation of adolescents and their families in the entire planning, development, and intervention of mobile apps is likely to contribute to more widely accepted and understood interventions by individuals living with chronic conditions in the future.

### Impact of Intervention on Parents, Carers, and Health Care Professionals

Parents and carers also play a significant part in promoting the development of adolescents’ personal management skills in chronic conditions [78], but parents may be less confident than adolescents in using technology [79]. Furthermore, given that this is a relatively underdeveloped area of adolescent health services, it can be difficult for those health professionals who are themselves unfamiliar with mobile phone and tablet apps to engage effectively with adolescents via these media [80,81]; future research may consider alternative ways of engaging busy clinicians in research, such as telephone interviews [82].

None of the studies reviewed specifically considered the impact of the technology on parents and their role in the development of adolescents’ self-management skills. Similarly, although the mutual benefits of participatory design for both end users and developers were highlighted in the papers, there was little discussion about the implications of app usage on care delivery for health professionals. In one study, there was a mandatory consultation with a health professional to discuss the app [56]. Aldiss et al noted that involvement of professionals during the development process was “the first step in embedding the system into practice” [57] and without this, collaborative and focused clinical care is unlikely to improve despite technical advances and innovations. Expertise in working with adolescents in this area is essential because of the need to consider adolescents’ development in the context of chronic conditions, both in terms of clinical care and involvement in research. Training in adolescent health care is not yet universal so it may be difficult for those professionals who have unmet training needs in this area to engage effectively with adolescents via these media [83]. Addressing professional concerns will be important to ensure efficacy of such interventions, for example, Frøisland et al [56] reported that before their study, the participating professionals expressed a fear that SMS text messaging would be overused, but these assumptions proved unfounded.

### Limitations

Due to resource limitations, this review excluded mental health conditions and learning disabilities and was only able to consider the three common chronic physical conditions reported in the included studies—asthma, cancer, and diabetes. There is, however, emerging data in the area of mental health care, albeit not specifically targeting adolescents, reporting that the majority of existing apps for mental health care lack scientific evidence about their efficacy [64,84]. In future, therefore, it will be important to reflect on the use of apps for adolescents with other chronic physical, as well as mental health, conditions, as many of the issues facing these adolescents may be similar.

While we used a recognized tool to assess the quality of the four studies, it did not consider factors such as user and health professional involvement in intervention, yet these factors are recognized as being increasingly important in the development and evaluation of complex interventions.

The small evidence base identified by our review emphasizes the need for future high-quality, sophisticated trials in the area of app development for adolescents with chronic conditions, and the total sample size of 46 participants limits generalizability of the findings. The dearth of existing evidence prevented us from commenting on the effectiveness of mobile apps designed to support adolescents’ management of their physical conditions, as had been the objective at the outset. This in itself was an important finding and generated stimulating discussion around what the next step should be, from a multi-professional expert audience at an international conference where preliminary findings from this review were reported [85]. A clear recommendation from this work is the need for high-quality RCTs in this field. Given the paucity of papers meeting the criteria for this review, it will be valuable to repeat the review and refine its original objectives in 2-3 years when more evidence is likely to exist.

Our rationale for using the WHO definition of young people aged 10-24 years was based on the specific developmental implications of this age group, which differ considerably from those of younger children and older adults; this meant that papers which were otherwise potentially relevant needed to be excluded. For example, of the 19 full-text papers considered for eligibility, the age range of reported populations for some studies fell outside our predetermined definition. Where we were unable to extract specific data that related to 10-24-year-old participants, either from the papers themselves or by communication with the original authors, we excluded those studies from our analysis. While it is outside the scope of this review to return to the search stage and revise the inclusion and exclusion criteria, it is a consideration for a future review.

Information on adolescents who decline to take part in studies is of major importance in research with this age group, particularly as research nonrespondents have been reported to have poorer health outcomes than those who do respond. In addition, identified barriers to the use of mobile technologies by adolescents, including the disparity of access to mobile devices and the potential for habituation, suggest that the use of IT to address health issues may be limited or even harmful to adolescents [27,28] None of the studies reviewed specifically considered these issues and it will be important for future studies to consider these factors, particularly in studies involving adults where habituation has already been highlighted as a potential limitation of individual apps [75]. Moreover, due to the lack of demographic details in the studies reviewed, comments regarding any gender differences in the use of apps are not possible. This is of interest in light of reports that adolescent females are more likely to access the Internet for health matters than adolescent males [74].

Recommendations from our systematic review emphasize the value of a multidisciplinary team enabling expert clinical and patient involvement in the app design, development, and evaluation stages, as well as the involvement of technological and research personnel. It is also recommended that future trials are based on sound theory and are tested across age groups (where appropriate, and while recognizing the different developmental stages of adolescence), gender, and ethnicity. Future work should also carefully consider which primary and secondary outcomes are important to assess, and the best medium- to long-term follow-up times in context of the longevity and persistence of any behavior change observed. Furthermore, it is anticipated that initially developing a robust, adolescent-friendly app in one condition may have the additional advantage of subsequent adaptability and/or transferability across other conditions.  

### Conclusions

In conclusion, a key finding of the review is the paucity of evidence-based apps that exist in contrast to the thousands of apps available on the app market that are not evidence-based or user and professional informed. Although we aimed to assess the effectiveness of apps, the dearth of studies meeting our criteria meant that we were unable to be conclusive in this regard. This review provides valuable findings and paves the way for future rigorous development and evaluation of health apps for adolescents with chronic conditions. There remains a need for a phased approach to well-designed trials of mobile phone and tablet apps which resonate with the lives of adolescents, that can be feasibly transferred into real-life settings and which involve adolescents, parents, and health professionals in their design, development, and evaluation. Based on the available evidence, apps may be considered feasible health interventions, but more studies involving larger sample sizes, and with patient and health professional input at all stages, are needed to determine apps’ acceptability and effectiveness.

## Appendix

Multimedia Appendix 1 Excluded studies and reasons for exclusion.

## Abbreviations

AAP: asthma action plan
ASyMS: Advanced Symptom Management System
ASyMS-YG: Advanced Symptom Management System for young people
CINAHL: Cumulative Index to Nursing and Allied Health Literature
Cond.: condition
EPHPP: Effective Public Health Practice Project
F: female
HbA1c: hemoglobin A1c (glycated hemoglobin)
ICC: intraclass correlation coefficient
ID: intervention duration
IMS: Institute of Medical Science
IMS Health: Institute of Medical Science Institute of Healthcare Informatics
IT: information technology
LSS-A: Life Situation Scale for Adolescents
N/A: not applicable
NHS: National Health Service
NIHR: National Institute for Health Research
PICOS: population or participant, intervention or indicator, comparator or control, outcome, and study design
PRISMA: Preferred Reporting Items for Systematic Reviews and Meta-Analyses
PROSPERO: international prospective register of systematic reviews
QOL: quality of life
RCT: randomized controlled trial
SMS: short message service
SS: sample size
STAI: State-Trait Anxiety Inventory
UMRI: University of Manchester Research Institute
WCDMA: wideband code-division multiple access
WHO: World Health Organization
