# Supplementary material for: Apps and Adolescents: A Systematic Review of Adolescents’ Use of Mobile Phone and Tablet Apps That Support Personal Management of Their Chronic or Long-Term Physical Conditions
Source: J Med Internet Res. 2015 Dec 23;17(12):e287. doi: 10.2196/jmir.5043 (PMC4704897; doi:10.2196/jmir.5043)
Supplement: Multimedia Appendix 1 [file jmir_v17i12e287_app1.pdf]

Multimedia Appendix 1. Excluded studies and reasons for exclusion.

Title	Key	Notes
ICanCope with Pain: User-centred design of a web and mobile-based self-management program for youth with chronic pain based on identified health care needs [40]	1	Type of study design
E-health: Stipulation of mobile phone technology in adolescent diabetic patient care [41]	2	Conference abstract
To WhatsApp or not to WhatsApp? What could be done with new social media to manage type 1 diabetes in adolescents? [42]	3	Conference abstract
Clinical results from transitional and home trials of outpatient closed-loop control [43]	4	Population intervention
Improved education and self-management in children and adolescents with asthma using a personalized smartphone application [44]	5	Population
Clinical and cost effectiveness of mobile phone supported self-monitoring of asthma: Multicentre randomised controlled trial [39]	6	Population intervention
Enhancing self-efficacy for self-management in people with cystic fibrosis [45]	7	Population intervention
Usability testing of a smartphone for accessing a web-based e-diary for self-monitoring of pain and symptoms in sickle cell disease [46]	8	Intervention
Utility of a wireless, handheld monitoring system in the management of hemophilia patients [47]	9	Population
Contracting and monitoring relationships for adolescents with type 1 diabetes: A pilot study [48]	10	Intervention
Facilitating self-management in adolescents and adults with cystic fibrosis: A pilot study [49]	11	Population
Cell phone intervention to improve adherence: Cystic fibrosis care team, patient, and parent perspectives [50]	12	Type of study design
Use of handheld wireless technology for a home-based sickle cell pain management protocol [51]	13	Population study design
Patients' perceptions and experiences of using a mobile phone-based advanced symptom management system (ASyMS) to monitor and manage chemotherapy related toxicity [52]	14	Population
The daily (Daily Automated Intensive Log for Youth) trial: A wireless, portable system to improve adherence and glycemic	15	Intervention

control in youth with diabetes [53]
